# Integrated bioinformatics analysis of the crucial candidate genes and pathways associated with glucocorticoid resistance in acute lymphoblastic leukemia

**DOI:** 10.1002/cam4.2934

**Published:** 2020-02-25

**Authors:** Yanxin Chen, Peifang Jiang, Jingjing Wen, Zhengjun Wu, Jiazheng Li, Yuwen Chen, Lingyan Wang, Donghui Gan, Yingyu Chen, Ting Yang, Minhui Lin, Jianda Hu

**Affiliations:** ^1^ Fujian Institute of Hematology Fujian Provincial Key Laboratory of Hematology Fujian Medical University Union Hospital Fuzhou China

**Keywords:** acute lymphoblastic leukemia, bioinformatic analysis, glucocorticoid resistance, MYC, signaling pathway

## Abstract

Glucocorticoids (GC) are the foundation of the chemotherapy regimen in acute lymphoblastic leukemia (ALL). However, resistance to GC is observed more frequently than resistance to other chemotherapy agents in patients with ALL relapse. Moreover, the mechanism underlying the development of GC resistance in ALL has not yet been fully uncovered. In this study, we used bioinformatic analysis methods to integrate the candidate genes and pathways participating in GC resistance in ALL and subsequently verified the bioinformatics findings with in vitro cell experiments. Ninety‐nine significant common differentially expressed genes (DEGs) associated with GC resistance were determined by integrating two gene profile datasets, including GC‐sensitive and ‐resistant samples. Using Kyoto Encyclopedia of Genes and Genomes (KEGG) and REACTOME pathways analysis, the signaling pathways in which DEGs were significantly enriched were clustered. The GC resistance‐related biologically functional interactions were visualized as DEG‐associated Protein–Protein Interaction (PPI) network complexes, with 98 nodes and 127 edges. MYC, a node which displayed the highest connectivity in all edges, was highlighted as the core gene in the PPI network. Increased C‐MYC expression was observed in adriamycin‐resistant BALL‐1/ADR cells, which we demonstrated was also resistant to dexamethasone. These results outlined a panorama in which the solitary and scattered experimental results were integrated and expanded. The potential promising target of the candidate pathways and genes involved in GC resistance of ALL was concomitantly revealed.

## Graphical abstract

These results outlined a panorama in which the solitary and scattered experimental results were integrated and extended. The potential promising target of the candidate pathways and genes in GC resistant acute lymphoblastic leukemia was concomitantly revealed.

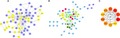

## 1 INTRODUCTION

Acute lymphoblastic leukemia comprises a group of aggressive aberrant hematopoietic malignancies, including acute/chronic lymphoblastic leukemia (ALL/CLL) and lymphoma. Combination chemotherapy regimen based on vincristine and prednisolone plus other classical chemotherapeutics and/or novel targeting agents is regarded as the essential care for patients with ALL.^1^, ^2^, ^3^ Although the advances in treatment strategies facilitated favorable clinical outcomes, a portion of patients with ALL unfortunately suffer single or multiple chemotherapeutic resistance and fall into relapse and/or refractory disease with dismal prognosis.^4^ Resistance to prednisolone, a glucocorticoid (GC) agent that is fundamental in the current ALL chemotherapy scheme, is more frequently observed than resistance to other drugs and hampers the possibility of prolonged survival.^5^

GC agonists inhibit leukemogenesis by binding the GC receptor and subsequently activating or suppressing transcription of target genes, which are related to various cellular processes such as cell cycle arrest and apoptosis.^6^, ^7^ Several studies have recently suggested candidate molecules and pathways that may be responsible for the development of GC resistance, including MLL‐rearrangement, and PI3K/AKT pathway and MAPK signaling cascades hyperactivation.^8^, ^9^, ^10^, ^11^, ^12^, ^13^, ^14^ Despite the increasing insights into several aspects of this mechanism, a comprehensive overview of the integrated biological landscape underlying GC resistance in ALL is currently missing.

Recently, high‐throughput sequencing and microarrays of human disease samples have generated massive bioinformatics data, which facilitated understanding the molecular mechanisms involved in the related biological process. In this study, we identified and integrated the common differentially expressed genes (DEGs) from two gene expression profiles of GC‐sensitive and ‐resistant ALL samples. Subsequently, we verified those bioinformatics findings with in vitro cell experiments. Our findings integrate individual experimental results from previous studies to illustrate the landscape of biological processes that lead to acquisition of GC resistance. Based on these results, novel treatment opportunities and therapeutic targets can be envisaged.

## 2 METHODS

### 2.1 Derivation of genetic data

Microarray datasets of gene expression profiles with the accession numbers http://www.ncbi.nlm.nih.gov/geo/query/acc.cgi?acc=GSE5820 and http://www.ncbi.nlm.nih.gov/geo/query/acc.cgi?acc=GSE19143, both unique, were downloaded from the NCBI‐gene expression omnibus (NCBI‐GEO) ( ://www.ncbi.nlm.nih.gov/geo/), a public functional genomics data repository.^15^, ^16^ The microarray of http://www.ncbi.nlm.nih.gov/geo/query/acc.cgi?acc=GSE5820 was performed on GPL96 Affymetrix Human Genome U133 platform and included 13 GC‐sensitive and 16 GC‐resistant samples (Submission date: Sep 13, 2006, last update date: Aug 10, 2018). Regarding the http://www.ncbi.nlm.nih.gov/geo/query/acc.cgi?acc=GSE19143 data, total RNA of 14 non‐infant GC‐sensitive and 13 non‐infant GC‐resistant samples were isolated and mRNA array was performed on GPL96 Affymetrix Human Genome U133A Array (Submission date: Nov 23, 2009, last update date: Aug 10, 2018). GC‐resistant samples were those with poor response to prednisolone, defined as failure to show effective cytoreduction after 7 days of GC therapy.^17^

### 2.2 Differentially expressed gene identification

Differentially expressed genes between GC‐sensitive and ‐resistant samples were identified by analyzing raw data with GEO2R on GEO database. A Log_2_ transformation was performed, and the Log_2_‐fold change (Log_2_FC) was calculated in the preprocessed microarray data derived from Series Matrix File on GEO database for integrated analysis. *P* < .05 and |Log_2_FC| > 1 was considered as the cut‐off criterion for significantly DEG screening.

### 2.3 Gene ontology and pathway enrichment analyses

GO enrichment analysis was performed using the DAVID (://david.ncifcrf.gov/conversion.jsp), a web‐based platform for gene functional annotations and biological meaning elucidation. Pathway analysis allows to understand molecular interactions in the pathway maps. Pathways enrichment analysis was generated by the Kyoto Encyclopedia of Genes and Genomes (KEGG) and REACTOME, which were included in the DAVID website, with *P < *.05 as the threshold value.

### 2.4 Integration of protein–protein interaction (PPI) network

String JAVA platform (http://string-db.org/), an online protein–association network platform, was utilized to expand the DEG‐encoded proteins and protein‐protein interaction (PPI) network. Further, the PPI network was imported into Cytoscape software for analyzing protein interaction links and modules, and accessing the interactions of the candidate DEG‐encoding proteins associated with GC resistance of ALL. Hub genes were identified by cytoHubba analysis in Cytoscape.

### 2.5 Hub gene validation and genetic alteration

The mRNA expression level of the hub genes was validated on the Oncomine database (://www.oncomine.org/resource/login.html). The genetic alterations of hub genes were investigated on the cBio Cancer Genomics Portal (://cbioportal.org).

### 2.6 GC resistance‐related miRNAs and predictions of miRNA targets

GC resistance‐related miRNAs were identified by searching for the terms “GC resistant AND miRNA” in PubMed. Based on the papers obtained by the search, hsa‐miR‐142‐3p and hsa‐miR‐17‐5p were selected as the miRNAs associated with GC resistance.^18^ Target genes were predicted by the miRNA‐target gene prediction databases miRwalk (http://mirwalk.umm.uni-heidelberg.de) and TargetScan (http://www.targetscan.org/vert_71/). The target genes authenticated by miRwalk and TargetScan were analyzed by intersection. Target genes‐miRNA interaction network was constructed using Cytoscape software.

### 2.7 Generation of target genes‐TFs interaction network

The genes which overlapped between GC‐resistant genes and human TFs (transcript factors) were considered the GC‐resistance TFs. The targeted genes were predicted on the Cistrome Data Browser (://cistrome.org/db/). The target genes‐TF interaction network was constructed using Cytoscape software.

### 2.8 Cell culture

Due to lack of dexamethasone‐sensitive and ‐resistant ALL cell lines, BALL‐1 and adriamycin (ADR)‐resistant cells BALL‐1/ADR were employed to evaluate dexamethasone sensitivity first and for subsequent experiments. Cells were preserved in our laboratory (Fujian Institute of Hematology, Fuzhou, China) and cultured in RPMI 1640 medium (Hyclone, UT, USA) supplemented with 10% heat‐inactivated fetal bovine serum (FBS, Gibco, UT) in a humidified incubator (Thermo, USA) maintained at 37°C with 5% CO_2_. Then, 0.5 μg/mL ADR (Sigma, MO) was added to BALL‐1 cell culture medium to maintain drug resistance. The medium was changed every other day. Cells in logarithmic growth phase were used in experiments. BALL‐1 cells were incubated in ADR‐free medium for 2 weeks for further experiments.

### 2.9 Dexamethasone sensitivity assay

BALL‐1 and BALL‐1/ADR cells were incubated with serially diluted dexamethasone solution ranging from 0 to 200 μg/mL for 72 hours. The 3‐(4,5‐dimethylthiazol‐2‐yl)‐2,5 diphenyltetra‐zolium bromide (MTT, Sigma, MO, USA) assay was performed to detect cell proliferation as previously described.^19^ Absorbance was measured with an Elx808 Absorbance Microplate spectrophotometer (BioTek, UT) at reference wavelengths of 490 and 630 nm. The proliferation inhibitory rate (%) was calculated by: [1‐(absorbance of experimental well/absorbance of control well)] × 100%.

### 2.10 Quantitative real‐time PCR (qPCR)

Total RNA was extracted using TRIzol reagent (Life Tech Invitrogen, CA, USA) and was subjected to reverse transcription with GoScript^TM^ Reverse Transcription System kit according to the manufacturer's protocol (Promega, Madison, USA). cDNA was amplified using SYBR Green PCR master mix (Life Tech Invitrogen, CA, USA). PCR was performed on ABI7500 Real Time PCR system (Applied Biosystems, CA, USA). Results were analyzed using the ∆CT and 2^−∆∆CT^ quantification method. The nucleotide sequences of the primers were as follows: 18s: sense: 5′‐GACACGGACAGGATTGACAGATTG‐3′, antisense: 5′‐TGCCAGAGTCTCGTTC.

GTTATCG‐3′; c‐myc: sense: 5′‐TCCTGGCAAAAGGTCAGAGT‐3′, anti‐sense: 5′‐TCTGACACTGTCCAACTTGAC‐3′.

### 2.11 Western blotting

Western blot analysis was performed as described in our previous study.^20^ The primary antibodies used for immunoblotting were GADPH and c‐myc (Cell Signaling Technology, Danvers, MA, USA), Immunoreactivity was detected by chemiluminescence reaction with an enhanced chemiluminescence (ECL) kit (Pierce/Thermo Fisher Scientific, Rockford, IL, USA).

### 2.12 Statistics

Experiments were performed in triplicate. Results of measurement data were expressed as means ± SEM Student's *t *test and one‐way analysis of variance (ANOVA) test were used to assess significant differences between groups according to data characteristics. Significance was set at a *P *< .05. All data were analyzed using SPSS statistics software 23.0 or GraphPad Prism Software 6.0.

## 3 RESULTS

### 3.1 Identification of GC resistance‐associated DEGs in ALL

**Figure 1:**
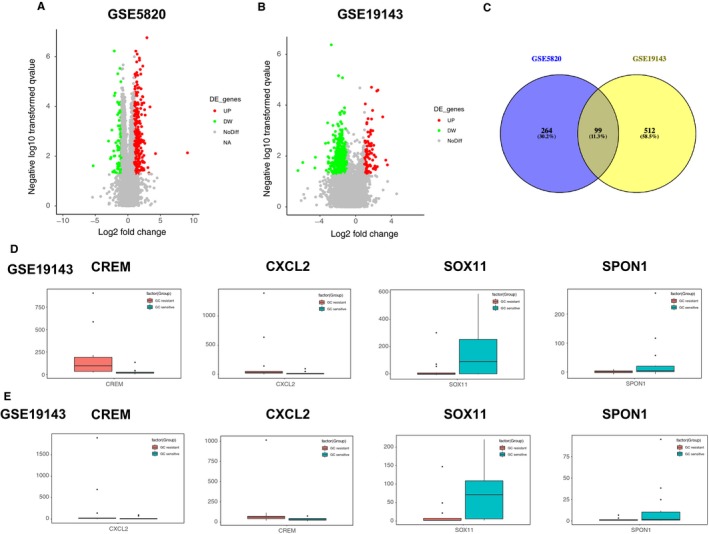
Identification of glucocorticoids (GC) resistance‐associated differentially expressed genes (DEGs) in acute lymphoblastic leukemia. Volcano plot of http://www.ncbi.nlm.nih.gov/geo/query/acc.cgi?acc=GSE5820 (A) and http://www.ncbi.nlm.nih.gov/geo/query/acc.cgi?acc=GSE19143 (B). Red color represents upregulated genes, green color represents downregulated genes. (C) Identification of 99 commonly changed GC resistance‐related DEGs from two cohort profile datasets and using Venny website (://bioinfogp.cnb.csic.es/tools/venny/index.html). Blue color areas represent http://www.ncbi.nlm.nih.gov/geo/query/acc.cgi?acc=GSE5820 dataset, yellow color areas represent http://www.ncbi.nlm.nih.gov/geo/query/acc.cgi?acc=GSE19143, and the cross area represents the commonly changed DEGs. (D, E) The boxplot of the top two common up‐ (CREM, CXCL2) and downregulated (SOX11, SPON1) genes

GC‐sensitive and ‐resistant ALL gene expression profiles http://www.ncbi.nlm.nih.gov/geo/query/acc.cgi?acc=GSE5820 and http://www.ncbi.nlm.nih.gov/geo/query/acc.cgi?acc=GSE19143 were downloaded from NCBI‐GEO. From the two above GSE datasets, 363 and 611 DEGs were extracted, respectively, with *P* < .05 and |Log_2_FC| > 1 as threshold values. DEGs are listed in Table 1. The volcano plot of the two GSE profiles was generated (Figure 1A,B). Subsequently, 99 common DEGs were screened out from the two datasets using integrated bioinformatics assay, which included 52 upregulated genes and 47 downregulated genes in GC‐resistant ALL samples compared with GC‐sensitive samples (Figure 1C; Table 1). The boxplots of the top two common upregulated (CREM, CXCL2) and downregulated (SOX11, SPON1) genes are shown in Figure 1D,E. To visualize the prominent different distribution of the 99 common DEGs, heat maps of the two GSE datasets were generated using Morpheus software (Figure S1).

**Table 1:** Commonly changed differentially expressed genes (DEGs) between glucocorticoids (GC) sensitive and resistant samples in acute lymphoblastic leukemia (ALL)

Common DEGs	Gene name
Up‐regulated (n = 52)	ELF4, CXCL11, CXCL8, SPAG9, SPRY1, CCND2, MAPK7, CHN2, PDE4B, CD58, MECP2, SLC2A3, TCF7L2, HSPA1B, HSPA1A, CPD, ADM, SLC2A14, CDC42EP3, NUDT6, SLC39A8, SRSF8, KPNA4, ACSL1, MYC, MCL1, MAFF, GCH1, RIPK2, TIMP1, DAPK1, EIF5, PRKAR2B, EMP1, STAB1, BCL2A1, FERMT2, ITGA6, SERPINE1, LPAR6, GNA13, ATF3, NUP50, PON2, NEU1, NR4A2, NR4A3, ZNF165, FOSL2, HCAR3, CREM, CXCL2
Down‐regulated (n = 47)	SPON1, SOX11, ID4, NRTN, S100A9, LHPP, MARCKS, S100A8, PTPRM, CHST2, PCDH9, LCN2, FGF12, SLIT3, OLFM4, HRK, RPL35A, IRS1, BASP1, PDGFRA, AEBP1, VASH2, RFWD3, ZNF611, TMCC1, IGLC1, ORAI2, PAX5, PRG2, TBL1X, SAC3D1, ADAM3B, RPL37A, MRPS12, RECQL5, HP, PACS1, BAHCC1, CD79B, GTSE1, RHOBTB1, HNRNPM, IQCK, TCF3, TMSB15B, TMSB15A, KIAA0226L

### 3.2 GO enrichment analysis

**Figure 2:**
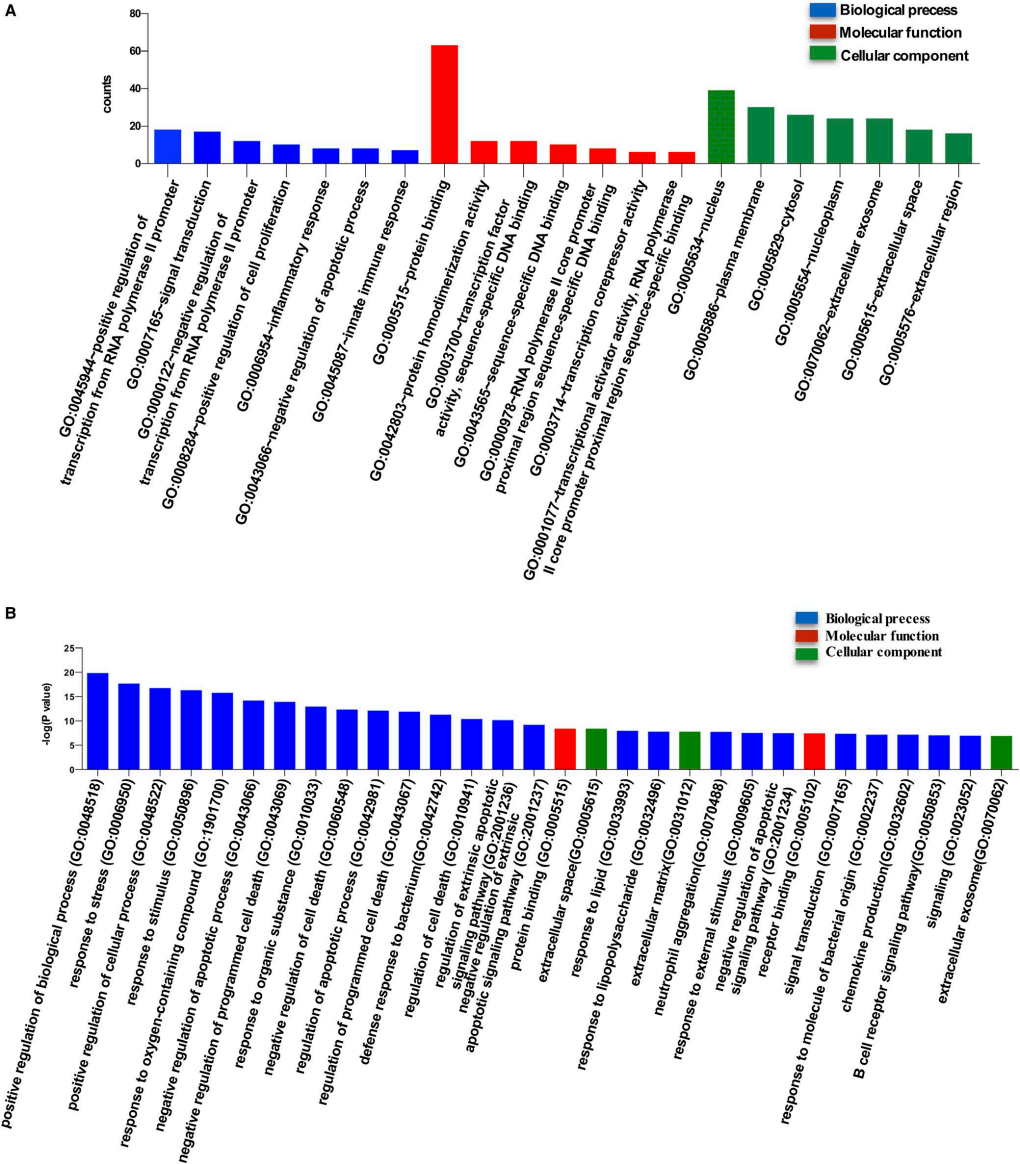
GO analysis. (A) GO analysis and significantly enriched GO terms of differentially expressed genes (DEGs) between glucocorticoids (GC)‐sensitive and ‐resistant acute lymphoblastic leukemia (ALL). Visualization of the significantly changed GO terms in the three functional groups. (B) Significantly enriched GO terms of DEGs in GC‐resistant ALL based on their functions. Differentially expressed genes functional enrichment was conducted using GO analysis on DAVID database

To understand the functional changes leading to GC resistance in ALL, the 99 candidate common DEGs were mapped on the DAVID database for GO analysis. As shown in Table 2, the primary GO terms enriched in upregulated DEGs were response to stimulus, positive regulation of biological process, and positive regulation of cellular process. Additionally, downregulated DEGs were predominantly enriched in negative regulation of transcription from RNA polymerase II promoter, defense response to bacterium, and innate immune response. Taken together, results of GO analysis suggested that the GC resistance‐associated DEGs were mainly centered in positive regulation of biological process, response to stress, and positive regulation of cellular process (−Log *P* value methods, Figure 2).

**Table 2:** GO analysis for up‐ and down‐regulated differentially expressed genes (top 5)

Term	Description	Gene count	*P* value
Up‐regulated			
GO:0 050 896	Response to stimulus	40	1.29E‐05
GO:0 048 518	Positive regulation of biological process	35	1.11E‐06
GO:0 048 522	Positive regulation of cellular process	32	9.49E‐06
GO:0 051 716	Cellular response to stimulus	31	2.75E‐02
GO:0 023 052	Signaling	29	8.13E‐03
Down‐regulated			
GO:0 000 122	Negative regulation of transcription from RNA polymerase II promoter	6	3.00E‐02
GO:0 042 742	defense response to bacterium	5	4.20E‐04
GO:0 045 087	Innate immune response	5	2.00E‐02
GO:0 006 412	Translation	4	2.40E‐02
GO:0 050 853	B cell receptor signaling pathway	3	7.70E‐03

### 3.3 Pathway enrichment analysis

Pathway enrichment analysis was performed to determine the candidate pathways connected to GC resistance‐associated DEGs and analyze their alterations. Downregulated DEGs were mainly enriched in “Ribosome pathway” in KEGG_PATHWAY analysis, and “Antigen activates B Cell Receptor (BCR) leading to generation of second messengers” and “RAF/MAP kinase cascade” in REACTOME_PATHWAY analysis. Upregulated DEGs were predominantly enriched in “Legionellosis,” “Transcriptional misregulation in cancer” and “Pathways in cancer” in KEGG_PATHWAY analysis, and “Viral RNP Complexes in the Host Cell Nucleus”, “Chemokine receptors bind chemokines,” and “Binding of TCF/LEF:CTNNB1 to target gene promoters” in REACTOME_PATHWAY analysis (Table 3).

**Table 3:** Significantly changed pathways in glucocorticoids (GC) resistant acute lymphoblastic leukemia (ALL)

Category	Term	*P* value	Genes	FDR
Down‐regulated DEGs				
KEGG	hsa03010:Ribosome	.058451099	RPL35A, MRPS12, RPL37A	43.24428955
REACTOME	R‐HSA‐983695:R‐HSA‐983695	.022857593	ORAI2, CD79B, IGLC1	21.28229012
REACTOME	R‐HSA‐5673001:R‐HSA‐5673001	.034409189	NRTN, PDGFRA, IRS1	30.39798248
Up‐regulated DEGs				
KEGG	hsa05134:Legionellosis	.001930947	CXCL2, CXCL8, HSPA1A, HSPA1B	2.122449312
KEGG	hsa05202:Transcriptional misregulation in cancer	.007090346	CCND2, BCL2A1, CXCL8, NR4A3, MYC	7.594024245
KEGG	hsa05200:Pathways in cancer	.008539708	GNA13, ITGA6, LPAR6, CXCL8, MYC, TCF7L2, DAPK1	9.080184539
KEGG	hsa05219:Bladder cancer	.015355087	CXCL8, MYC, DAPK1	15.78132383
KEGG	hsa04621:NOD‐like receptor signaling pathway	.027619574	CXCL2, CXCL8, RIPK2	26.71925927
KEGG	hsa04390:Hippo signaling pathway	.032309078	CCND2, SERPINE1, MYC, TCF7L2	30.54775066
KEGG	hsa04151:PI3K‐Akt signaling pathway	.074007029	ITGA6, MCL1, CCND2, LPAR6, MYC	57.40416186
KEGG	hsa05145:Toxoplasmosis	.092188264	ITGA6, HSPA1A, HSPA1B	65.81931717
REACTOME	R‐HSA‐168330:R‐HSA‐168330	.008577044	HSPA1A, HSPA1B	9.166509374
REACTOME	R‐HSA‐380108:R‐HSA‐380108	.026397272	CXCL2, CXCL8, CXCL11	25.81297847
REACTOME	R‐HSA‐4411364:R‐HSA‐4411364	.029707264	MYC, TCF7L2	28.57986805
REACTOME	R‐HSA‐3371453:R‐HSA‐3371453	.036177342	NUP50, HSPA1A, HSPA1B	33.71879102
REACTOME	R‐HSA‐3371568:R‐HSA‐3371568	.058553978	HSPA1A, HSPA1B	49.00493226
REACTOME	R‐HSA‐3371571:R‐HSA‐3371571	.078645388	HSPA1A, HSPA1B	59.91663333
REACTOME	R‐HSA‐418594:R‐HSA‐418594	.088167851	CXCL2, CXCL8, CXCL11, HCAR3	64.30509097

### 3.4 Key candidate pathways identification with DEGs PPI network

**Figure 3:**
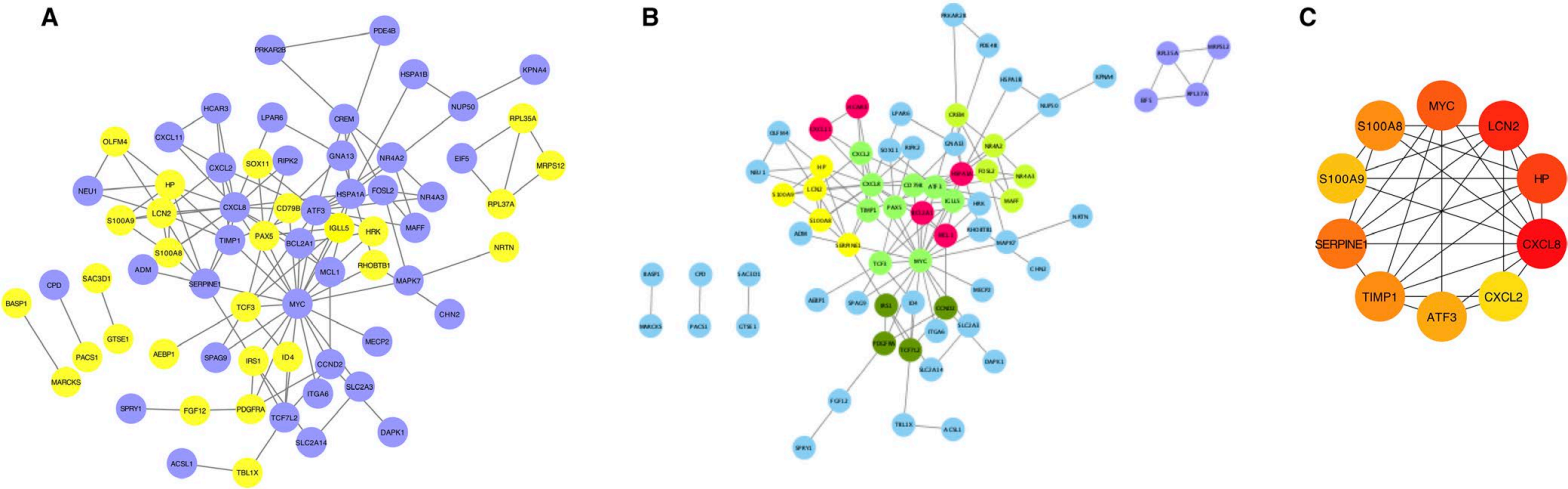
Differentially expressed genes (DEGs) protein–protein interaction (PPI) network complex based on String website and integrated by Cytoscape software. In this picture, each circle represents a gene (node) and each connection represents a direct or indirect connection (edge). (A) Protein–Protein Interaction network marked for separating up‐ and downregulated DEGs. Yellow color represents upregulated genes, and blue color represents downregulated genes. (B) Modules analysis in PPI network. Clusters were extracted by MCODE and presented with different colors. (C) The top 10 of genes with highest degrees identified by cytoHubba analysis. MYC displayed the highest connectivity in all interactions

String database and Cytoscape software were employed to identify and visualize the PPI network derived from candidate DEGs. A total of 98 out of the 99 common DEGs were inserted in the GC resistance‐associated PPI network, which comprised 98 nodes and 127 edges (molecular interaction) with PPI enrichment *P* value of 9.84E‐13, indicating a reliable GC‐resistant PPI network (Figure 3A). The topological properties are listed in Table S1. By Cytoscape MCODE analysis, 6 significant modules were screened from the PPI network (Figure 3B). Of these, module 1 included 5 nodes and 8 edges, and module 2 included 4 nodes and 5 edges. Using cytoHubba analysis, among the 98 nodes, MYC, CXCL8, ATF3, the three central node genes with degree > 10, were considered as hub genes in the PPI network. MYC, for which the nodes showed the highest connectivity in all interactions, was highlighted as the core gene connected to GC resistance‐related PPI network (Figure 3C).

### 3.5 Target genes‐miRNA interaction and TF‐target genes network

**Figure 4:**
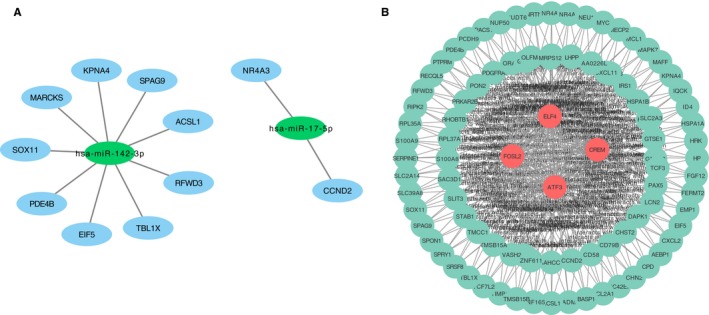
The target genes‐miRNA network and the transcript factors (TF)‐target genes network. (A) Target genes‐miRNA network integrated by Cytoscape software. Green color represents miRNA. Blue color represents target genes. (B) Green color represents results integrated by Cytoscape software. Red color represents TFs. Green color represents target genes

The 64 and 194 target genes of hsa‐miR‐142‐3p and hsa‐miR‐17‐5p, respectively, were verified using the two databases miRwalk and TargetScan (Table S2). The genes overlapping between DEGs and hsa‐miR‐142‐3p were MARCKS, EIF5, SPAG9, ACSL1, KPNA4, PDE4B, RFWD3, TBL1X, and SOX11. The genes overlapping between DEGs and hsa‐miR‐17‐5p were NR4A3 and CCND2. Then, the target genes‐miRNA network was constructed using Cytoscape software (Figure 4A).

CREM, ATF3, ELF4, and FOSL2, the four genes overlapping between TFs and GC‐resistant DEGs, were subjected to target gene prediction. Table S3 lists the genes overlapping between the target genes of the TFs and DEGs. The generated TF‐target genes network is shown in Figure 4B.

### 3.6 Validation and genetic alteration of hub Genes

**Figure 5:**
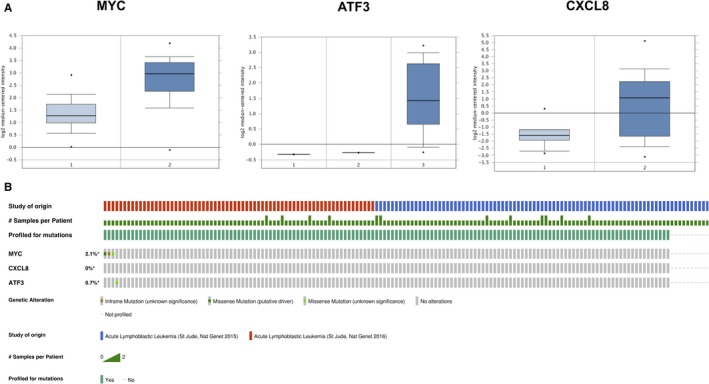
Validation and genetic alteration of hub genes. (A) mRNA expression of MYC, ATF3, and CXCL8 in acute lymphoblastic leukemia (ALL) samples and normal samples on Oncomine database. MYC, CXCL8, and ATF3 exhibited a 2.564, 4.446, 3.605‐fold change, respectively, between lymphoblastic leukemia and normal samples. (B) Genetic alterations in the ALL samples. About 0% to 2.1% of alterations for the 3 queried genes were calculated in the examined ALL studies. Genetic mutations of MYC and ATF3 were 2.1% and 0.7% respectively. No CXCL8 alteration was observed

MYC, CXCL8, and ATF3 were considered the hub genes and queried on Oncomine database and cBio portal platform to investigate their gene expression and genetic alterations in lymphoblastic leukemia. The mRNA expression level of MYC, CXCL8 and ATF3 exhibited a 2.564, 4.446, 3.605‐fold change, respectively, between lymphoblastic leukemia and normal samples in the examined study^21^, ^22^ (Figure 5A). The alterations for the 3 queried genes were calculated to be between 0% and 2.1% in the examined ALL samples. Genetic mutations of MYC and ATF3 were 2.1% and 0.7%, respectively. No CXCL8 alteration was observed in the examined study^23^ (Figure 5B).

### 3.7 Confirmation of C‐MYC by in vitro cell experiments

For confirmation, we first evaluated cytotoxicity of dexamethasone in BALL‐1 and BALL‐1/ADR cells. Using MTT assay, we observed markedly lower dexamethasone sensitivity in BALL‐1/ADR cells than in the parental cells BALL‐1. The IC50 of dexamethasone in BALL‐1 cells and BALL‐1/ADR cells was 5.57 ± 1.32 μg/mL and 32.40 ± 1.33 μg/mL, respectively.

**Figure 6:**
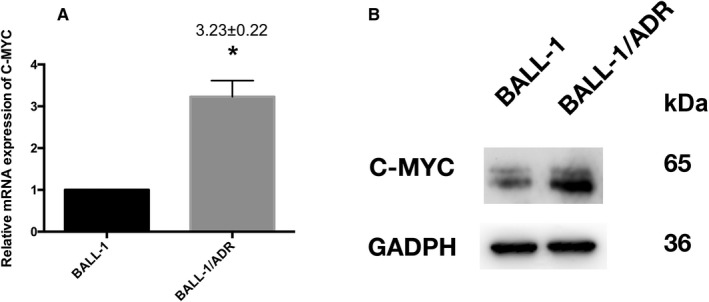
Validation of C‐MYC. (A) quantitative real‐time‐PCR was performed to examine the expression of C‐MYC mRNA. Ribosomal RNA 18S was used as an internal control and for normalization of the data. Upregulation of C‐MYC mRNA was observed in BALL‐1/ADR cells, with 2^−∆∆Ct^ values equal to 3.23 ± 0.22, relative to parental control cells (2^−∆∆Ct^ equal to 1). (B) Detection of the C‐MYC protein

Based on the MTT results, we examined C‐MYC expression at the mRNA and protein level using qRT‐PCR and western blot (Figure 6A,B). A significantly increased expression of C‐MYC mRNA and protein in BALL‐1/ADR cells was observed.

Taken together, using MTT, qRT‐PCR, and western blot assays, we validated the potential molecular mechanism of GC resistance in lymphoid tumor cells.

## 4 DISCUSSION

Despite the reassuring achievements in the treatment of patients with ALL, resistance to GC agonists has been a major obstacle in eliminating leukemia cells and maximizing cure.^24^ Several studies on GC resistance have elucidated that GC‐stimulated apoptosis is not induced in leukemia cells with poor response to GC agents.^25^, ^26^ Recently published articles partly expanded our understanding about the molecular mechanism of GC resistance, wherein the role of some molecules and signaling pathways including MLL rearrangement and JAK/SAT pathway alteration were determined. Nevertheless, limited by scattered information obtained from individual studies, a comprehensive internal mechanistic basis underlying the development of GC resistance remains to be uncovered.

In this study, we generated an integrated, biologically functional landscape of GC resistance in ALL using bioinformatic analysis. First, a total of 99 common DEGs (52 upregulated and 47 downregulated) were identified between GC‐sensitive and ‐resistant samples by integrating two gene profile datasets from two different researches and analyzing the datasets with bioinformatic methods. Further, the 99 candidate DEGs were enriched and categorized into 3 groups: biological process, molecular functions, and cellular component terms using GO functional analysis. Finally, DEG‐related PPI network was expanded, and 98 nodes were identified with 127 edges, of which one node exhibited the highest connectivity in all edges and was regarded as the core gene in the PPI network.

The upregulated DEGs were mainly enriched in the BP terms related to response to stimulus, positive regulation of biological process, and positive regulation of cellular process. The downregulated DEGs were instead mainly enriched in BP terms connected with negative regulation of transcription from RNA polymerase II promoter, defense response to bacterium, and innate immune response.

Transcriptional misregulation in cancer (map05202 on KEGG database: ://www.genome.jp/kegg/pathway.html) pathway was one of the top pathways identified, which derived from multiple TF alterations, including genes fusion, amplification, mutation, deletion, rearrangements and translocation, resulting in various oncogenic processes such as differentiation resistance, inhibition of apoptosis, and cellular invasion. Chromosomal translocations and rearrangements of mixed lineage leukemia (MLL) gene, which is located at 11q23, are frequently represented as t (4;11) (q21; q23), t (11;19) (q23; p13.3) and t (9;11) (p22; q23), result in fusion proteins and subsequently lead to aberrant gene expression associated with leukemogenesis and chemotherapeutics resistance. Patients with ALL bearing MLL‐rearrangements show poor survival also due to cellular resistance to various chemotherapeutics, especially to GC, which is in accordance with the results of KEGG analysis.^27^ Notably, CCND2 is upregulated and hyperactivated by the amplification of transcription factors with oncogene properties, and thereby accelerates cell cycle by promoting G1/S phase transformation and increasing cell proliferation, which is considered the critical oncogenesis process in leukemia and lymphoma. Real et al proposed that upregulation of CCND2 is involved in the protection against Gamma‐secretase inhibitors (GSIs), which are Notch pathway inhibitors used in GC‐resistant T‐cell acute lymphoblastic leukemia (T‐ALL).^28^ Additionally, reliability of the top enriched pathway associated with GC resistance, Transcriptional misregulation in cancer, is further supported by studies reporting that BCL2A1, PAX5, and CXCL8 play important roles in GC‐resistant ALL.^29^, ^30^ Moreover, Pathways in cancer (map05200 on KEGG website) comprised various oncogenesis signaling pathways including MAPK, JAT/STAT, and PI3K‐AKT signaling pathways, which were confirmed to promote development of GC resistance in leukemia by multiple independent studies.^7^, ^8^, ^19^, ^31^, ^32^ Collectively, results of enriched KEGG pathway analysis were positively correlated with experimental findings. However, further investigations are needed to explore and confirm the potentially significant pathways for GC resistance in ALL and to achieve a comprehensive understanding of this process.

MYC, which was identified as the core gene in GC resistance‐associated PPI network and is the node that showed the highest connectivity degrees therein, is also a modulator of numerous cell processes by transcriptional regulation of its target genes. We did not perform survival analysis due to lack of data on ALL survival in the two gene expression profiles, in the TCGA, and in Oncomine database. However, the role of MYC in lymphoid malignancy was elucidated. MYC upregulation, caused by deregulated activity of MYC transcriptional network, enhances cancer proliferation and cellular drug resistance according to recent researches. Schubbert S et al revealed that targeting MYC and PI3K pathway could eliminate leukemia‐initiating cells in T‐ALL.^33^ Moyo et al showed that MYC is overexpressed in pre‐malignant B cells with activation of PI3K/AKT signaling pathway. Moreover, MYC overexpression promoted resistance to Btk inhibition in B cell malignancy.^34^ Notably, the GC resistance‐promoting role of MYC in ALL was firstly elucidated by Renner K.^35^ Han et al revealed that MYC displays constitutive activation in B‐ALL cell lines, especially GC‐resistant B‐ALL, indicating the critical role of MYC in evolution of GC resistance.^26^ The positive impact of MYC on progression of GC resistance in ALL was subsequently strengthened by other studies.^36^, ^37^ Hence, the results of PPI network were in accordance with experimental results on GC resistance in ALL. The role of multiple genes in the network were determined, such as MYC, MCL1 and CCND2, which expanded our knowledge on development of GC resistance in ALL.

In this study, to validate the reliability and applicability of bioinformatics analysis, we investigated C‐MYC in BALL‐1 and BALL‐1/ADR cells, which displayed dexamethasone sensitivity and resistance, respectively. Increased expression of C‐MYC was observed in BALL‐1/ADR cells, which is consistent with bioinformatics analysis results.

In summary, bioinformatics analysis was used to identify the biological networks and in vitro experiments were performed to verify the bioinformatics findings. Nevertheless, validation in a larger group of patients and in‐depth experiments in vivo and in vitro are needed to improve and examine the accuracy of the bioinformatics analysis.

Taken together, the bioinformatic analysis employed here proved to be a convenient and valid tool that offered us a novel and systematic insight into the mechanism of GC resistance in ALL. Several pathways were identified which are potentially involved in GC resistance in ALL. Importantly, our data revealed a comprehensive interaction network which highlighted MYC as a hub gene among various candidate genes. Our findings integrate individual experimental results from previous studies to illustrate the landscape of biological processes that lead to acquisition of GC resistance. Based on these results, novel treatment opportunities and therapeutic targets can be envisaged.

## CONFLICT OF INTERESTS

There are no conflict of interest to disclose.

## AUTHORS' CONTRIBUTION

Jianda Hu, Yanxin Chen conceived and designed the experiment; Peifang Jiang, Jingjing Wen, Donghui Gan and Yingyu Chen operated the experiment, Jiazheng Li, Yuwen Chen, Ting Yang analyzed the data, Yanxin Chen, Liangyan Wang, Zhengjun Wu and Jianda Hu wrote the paper. Ting Yang, Minhui Lin and Jianda Hu reviewed the paper.

## ETHICS APPROVAL AND CONSENT TO PARTICIPATE

This article does not contain any studies with human participants or animals performed by any of the authors.

## Data Availability

The data that support the findings of this study are available from the corresponding author upon reasonable request.
