# Supplementary material for: Integrated bioinformatics analysis of the crucial candidate genes and pathways associated with glucocorticoid resistance in acute lymphoblastic leukemia
Source: Cancer Med. 2020 Feb 25;9(8):2918–29. doi: 10.1002/cam4.2934 (PMC7163086; doi:10.1002/cam4.2934)
Supplement: Supplementary file 1 [file CAM4-9-2918-s001.pdf]

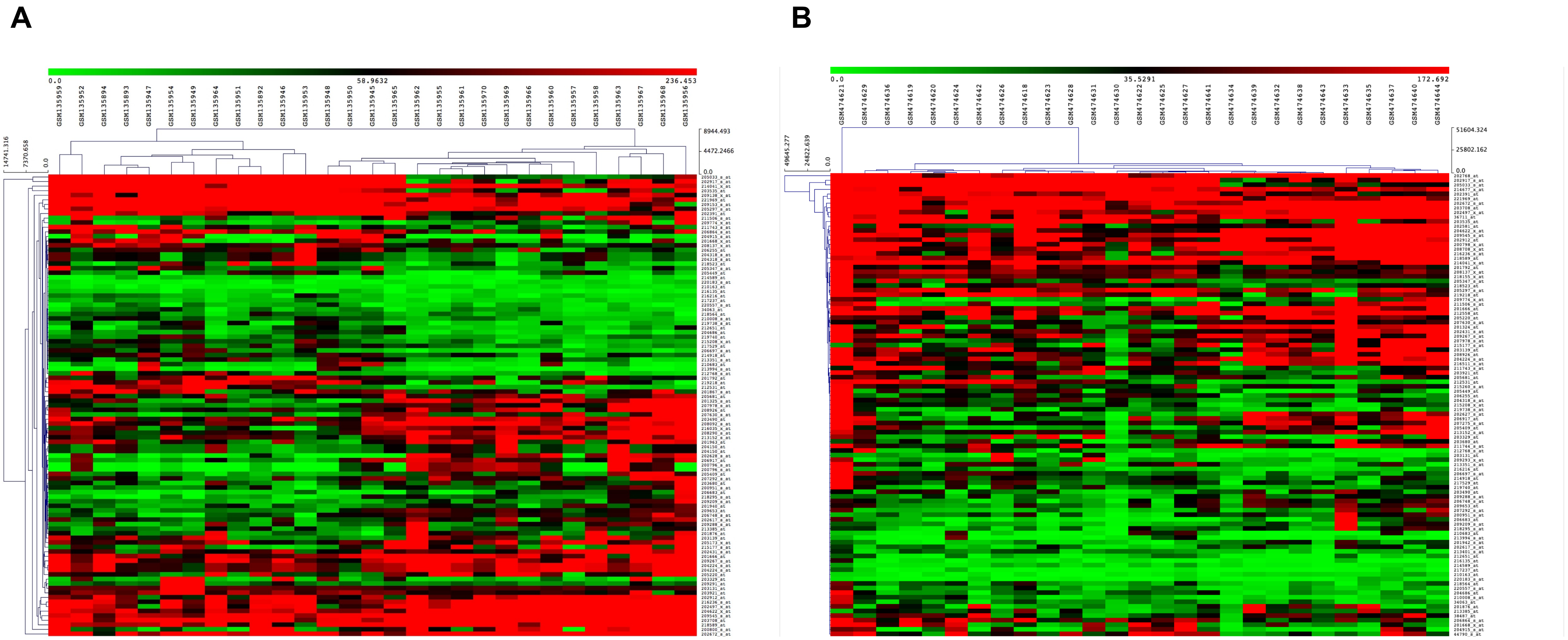


**Supplement figure1: Heatmap visualization of the DEGs between GC sensitivity and resistant samples of ALL from dataset GSE5820(A) and GSE19143(B).** Red area represented the up-regulated genes, green area represented the down-regulated genes.
