# Supplementary material for: Integrated bioinformatics analysis of the crucial candidate genes and pathways associated with glucocorticoid resistance in acute lymphoblastic leukemia
Source: Cancer Med. 2020 Feb 25;9(8):2918–29. doi: 10.1002/cam4.2934 (PMC7163086; doi:10.1002/cam4.2934)
Supplement: Supplementary file 2 [file CAM4-9-2918-s002.docx]

**Supplement Tables**

Supplement Table1. Basic information of the topological properties.

| #node1 | node2 | node1_string_internal_id | node2_string_internal_id | node1_external_id | node2_external_id | neighborhood_on_chromosome | phylogenetic_cooccurrence | homology | coexpression | experimentally_determined_interaction | database_annotated | automated_textmining | combined_score |
| --- | --- | --- | --- | --- | --- | --- | --- | --- | --- | --- | --- | --- | --- |
| S100A9 | S100A8 | 4443166 | 4443164 | 9606.ENSP00000357727 | 9606.ENSP00000357722 | 0 | 0 | 0.801 | 0.995 | 0.877 | 0.9 | 0.882 | 0.999 |
| RPL35A | RPL37A | 4449013 | 4448949 | 9606.ENSP00000419117 | 9606.ENSP00000418082 | 0 | 0 | 0 | 0.988 | 0.96 | 0.9 | 0.747 | 0.999 |
| CXCL2 | CXCL8 | 4449392 | 4438229 | 9606.ENSP00000427279 | 9606.ENSP00000306512 | 0 | 0 | 0.874 | 0.824 | 0 | 0.9 | 0.767 | 0.983 |
| HSPA1A | HSPA1B | 4444600 | 4444599 | 9606.ENSP00000364802 | 9606.ENSP00000364801 | 0 | 0.449 | 0.988 | 0.139 | 0.8 | 0.9 | 0.539 | 0.981 |
| MYC | TCF7L2 | 4451407 | 4443308 | 9606.ENSP00000479618 | 9606.ENSP00000358404 | 0 | 0 | 0 | 0 | 0.328 | 0.9 | 0.715 | 0.979 |
| CXCL2 | CXCL11 | 4449392 | 4438264 | 9606.ENSP00000427279 | 9606.ENSP00000306884 | 0 | 0 | 0 | 0.107 | 0.232 | 0.9 | 0.717 | 0.978 |
| SERPINE1 | TIMP1 | 4433266 | 4433052 | 9606.ENSP00000223095 | 9606.ENSP00000218388 | 0 | 0 | 0 | 0.174 | 0 | 0.9 | 0.712 | 0.974 |
| CXCL11 | CXCL8 | 4438264 | 4438229 | 9606.ENSP00000306884 | 9606.ENSP00000306512 | 0 | 0 | 0 | 0.107 | 0 | 0.9 | 0.678 | 0.968 |
| IGLL5 | CD79B | 4449581 | 4446076 | 9606.ENSP00000431254 | 9606.ENSP00000376544 | 0 | 0 | 0 | 0.341 | 0 | 0.9 | 0.478 | 0.962 |
| LCN2 | HP | 4444080 | 4441808 | 9606.ENSP00000362108 | 9606.ENSP00000348170 | 0 | 0 | 0 | 0.064 | 0 | 0.9 | 0.519 | 0.951 |
| LCN2 | OLFM4 | 4444080 | 4433058 | 9606.ENSP00000362108 | 9606.ENSP00000219022 | 0 | 0 | 0 | 0.239 | 0 | 0.9 | 0.399 | 0.95 |
| TCF7L2 | TBL1X | 4443308 | 4433034 | 9606.ENSP00000358404 | 9606.ENSP00000217964 | 0 | 0 | 0 | 0.063 | 0 | 0.9 | 0.37 | 0.935 |
| MCL1 | BCL2A1 | 4443243 | 4435793 | 9606.ENSP00000358022 | 9606.ENSP00000267953 | 0 | 0 | 0.653 | 0.062 | 0 | 0.9 | 0.772 | 0.928 |
| RPL35A | EIF5 | 4449013 | 4432973 | 9606.ENSP00000419117 | 9606.ENSP00000216554 | 0 | 0 | 0 | 0.262 | 0 | 0.9 | 0 | 0.923 |
| GNA13 | RHOBTB1 | 4448134 | 4440888 | 9606.ENSP00000400717 | 9606.ENSP00000338671 | 0 | 0 | 0 | 0.061 | 0.185 | 0.9 | 0.086 | 0.92 |
| RPL37A | EIF5 | 4448949 | 4432973 | 9606.ENSP00000418082 | 9606.ENSP00000216554 | 0 | 0 | 0 | 0.184 | 0 | 0.9 | 0.087 | 0.919 |
| PRKAR2B | CREM | 4435635 | 4435595 | 9606.ENSP00000265717 | 9606.ENSP00000265372 | 0 | 0 | 0 | 0.061 | 0 | 0.9 | 0.166 | 0.914 |
| HCAR3 | CXCL8 | 4449793 | 4438229 | 9606.ENSP00000436714 | 9606.ENSP00000306512 | 0 | 0 | 0 | 0.172 | 0 | 0.9 | 0.046 | 0.914 |
| RHOBTB1 | CHN2 | 4440888 | 4433253 | 9606.ENSP00000338671 | 9606.ENSP00000222792 | 0 | 0 | 0 | 0 | 0.096 | 0.9 | 0.094 | 0.911 |
| MAPK7 | NRTN | 4438648 | 4437881 | 9606.ENSP00000311005 | 9606.ENSP00000302648 | 0 | 0 | 0 | 0 | 0 | 0.9 | 0.099 | 0.906 |
| HCAR3 | CXCL2 | 4449793 | 4449392 | 9606.ENSP00000436714 | 9606.ENSP00000427279 | 0 | 0 | 0 | 0.098 | 0 | 0.9 | 0 | 0.905 |
| MCL1 | HRK | 4443243 | 4434556 | 9606.ENSP00000358022 | 9606.ENSP00000257572 | 0 | 0 | 0 | 0 | 0.613 | 0 | 0.765 | 0.905 |
| HCAR3 | CXCL11 | 4449793 | 4438264 | 9606.ENSP00000436714 | 9606.ENSP00000306884 | 0 | 0 | 0 | 0.076 | 0 | 0.9 | 0.054 | 0.904 |
| TCF3 | SPAG9 | 4435174 | 4434990 | 9606.ENSP00000262965 | 9606.ENSP00000262013 | 0 | 0 | 0 | 0 | 0 | 0.9 | 0.086 | 0.904 |
| HP | OLFM4 | 4441808 | 4433058 | 9606.ENSP00000348170 | 9606.ENSP00000219022 | 0 | 0 | 0 | 0 | 0 | 0.9 | 0.073 | 0.903 |
| ACSL1 | TBL1X | 4449224 | 4433034 | 9606.ENSP00000422607 | 9606.ENSP00000217964 | 0 | 0 | 0 | 0.062 | 0 | 0.9 | 0.058 | 0.903 |
| NEU1 | HP | 4444596 | 4441808 | 9606.ENSP00000364782 | 9606.ENSP00000348170 | 0 | 0 | 0 | 0 | 0 | 0.9 | 0.065 | 0.902 |
| SLC2A14 | SLC2A3 | 4450179 | 4432652 | 9606.ENSP00000445929 | 9606.ENSP00000075120 | 0 | 0.449 | 0.986 | 0.815 | 0.485 | 0 | 0.789 | 0.902 |
| NEU1 | OLFM4 | 4444596 | 4433058 | 9606.ENSP00000364782 | 9606.ENSP00000219022 | 0 | 0 | 0 | 0 | 0 | 0.9 | 0 | 0.9 |
| HSPA1B | NUP50 | 4444599 | 4441567 | 9606.ENSP00000364801 | 9606.ENSP00000345895 | 0 | 0 | 0 | 0.05 | 0 | 0.9 | 0 | 0.9 |
| HSPA1A | NUP50 | 4444600 | 4441567 | 9606.ENSP00000364802 | 9606.ENSP00000345895 | 0 | 0 | 0 | 0.05 | 0 | 0.9 | 0 | 0.9 |
| NEU1 | LCN2 | 4444596 | 4444080 | 9606.ENSP00000364782 | 9606.ENSP00000362108 | 0 | 0 | 0 | 0 | 0 | 0.9 | 0 | 0.9 |
| ATF3 | CREM | 4441415 | 4435595 | 9606.ENSP00000344352 | 9606.ENSP00000265372 | 0 | 0 | 0 | 0.078 | 0 | 0.8 | 0.506 | 0.9 |
| RPL35A | MRPS12 | 4449013 | 4447184 | 9606.ENSP00000419117 | 9606.ENSP00000384952 | 0 | 0 | 0 | 0.654 | 0.713 | 0 | 0.069 | 0.899 |
| RPL37A | MRPS12 | 4448949 | 4447184 | 9606.ENSP00000418082 | 9606.ENSP00000384952 | 0 | 0 | 0 | 0.654 | 0.709 | 0 | 0.061 | 0.897 |
| BCL2A1 | HRK | 4435793 | 4434556 | 9606.ENSP00000267953 | 9606.ENSP00000257572 | 0 | 0 | 0 | 0 | 0.613 | 0 | 0.719 | 0.886 |
| PAX5 | TCF3 | 4442079 | 4435174 | 9606.ENSP00000350844 | 9606.ENSP00000262965 | 0 | 0 | 0 | 0.053 | 0.091 | 0 | 0.868 | 0.876 |
| MYC | MCL1 | 4451407 | 4443243 | 9606.ENSP00000479618 | 9606.ENSP00000358022 | 0 | 0 | 0 | 0.065 | 0.125 | 0 | 0.84 | 0.858 |
| MYC | CCND2 | 4451407 | 4434845 | 9606.ENSP00000479618 | 9606.ENSP00000261254 | 0 | 0 | 0 | 0 | 0 | 0 | 0.821 | 0.821 |
| CXCL8 | TIMP1 | 4438229 | 4433052 | 9606.ENSP00000306512 | 9606.ENSP00000218388 | 0 | 0 | 0 | 0.119 | 0 | 0 | 0.795 | 0.812 |
| MYC | MAPK7 | 4451407 | 4438648 | 9606.ENSP00000479618 | 9606.ENSP00000311005 | 0 | 0 | 0 | 0 | 0.379 | 0 | 0.677 | 0.791 |
| MAFF | ATF3 | 4441516 | 4441415 | 9606.ENSP00000345393 | 9606.ENSP00000344352 | 0 | 0 | 0 | 0.359 | 0.363 | 0 | 0.526 | 0.79 |
| MARCKS | BASP1 | 4451269 | 4439274 | 9606.ENSP00000478061 | 9606.ENSP00000319281 | 0 | 0 | 0 | 0.155 | 0 | 0 | 0.76 | 0.789 |
| MYC | PAX5 | 4451407 | 4442079 | 9606.ENSP00000479618 | 9606.ENSP00000350844 | 0 | 0 | 0 | 0 | 0.087 | 0 | 0.753 | 0.765 |
| MYC | CXCL8 | 4451407 | 4438229 | 9606.ENSP00000479618 | 9606.ENSP00000306512 | 0 | 0 | 0 | 0.062 | 0 | 0 | 0.721 | 0.727 |
| S100A8 | CXCL8 | 4443164 | 4438229 | 9606.ENSP00000357722 | 9606.ENSP00000306512 | 0 | 0 | 0 | 0.189 | 0 | 0 | 0.669 | 0.72 |
| CXCL8 | SERPINE1 | 4438229 | 4433266 | 9606.ENSP00000306512 | 9606.ENSP00000223095 | 0 | 0 | 0 | 0.137 | 0 | 0 | 0.678 | 0.71 |
| TCF3 | AEBP1 | 4435174 | 4433280 | 9606.ENSP00000262965 | 9606.ENSP00000223357 | 0 | 0 | 0 | 0 | 0.379 | 0 | 0.548 | 0.707 |
| MYC | ATF3 | 4451407 | 4441415 | 9606.ENSP00000479618 | 9606.ENSP00000344352 | 0 | 0 | 0 | 0.096 | 0 | 0 | 0.676 | 0.695 |
| IRS1 | PDGFRA | 4438085 | 4434540 | 9606.ENSP00000304895 | 9606.ENSP00000257290 | 0 | 0 | 0 | 0.062 | 0 | 0.6 | 0.215 | 0.679 |
| ATF3 | SOX11 | 4441415 | 4439535 | 9606.ENSP00000344352 | 9606.ENSP00000322568 | 0 | 0 | 0 | 0 | 0.065 | 0 | 0.67 | 0.679 |
| PAX5 | SOX11 | 4442079 | 4439535 | 9606.ENSP00000350844 | 9606.ENSP00000322568 | 0 | 0 | 0 | 0 | 0.064 | 0 | 0.659 | 0.667 |
| CD79B | PAX5 | 4446076 | 4442079 | 9606.ENSP00000376544 | 9606.ENSP00000350844 | 0 | 0 | 0 | 0.255 | 0 | 0 | 0.57 | 0.666 |
| HSPA1A | CXCL8 | 4444600 | 4438229 | 9606.ENSP00000364802 | 9606.ENSP00000306512 | 0 | 0 | 0 | 0 | 0 | 0 | 0.652 | 0.652 |
| ATF3 | FOSL2 | 4441415 | 4435485 | 9606.ENSP00000344352 | 9606.ENSP00000264716 | 0 | 0 | 0.777 | 0.417 | 0.332 | 0 | 0.564 | 0.643 |
| MCL1 | CXCL8 | 4443243 | 4438229 | 9606.ENSP00000358022 | 9606.ENSP00000306512 | 0 | 0 | 0 | 0.069 | 0 | 0 | 0.625 | 0.636 |
| GNA13 | LPAR6 | 4448134 | 4445053 | 9606.ENSP00000400717 | 9606.ENSP00000367691 | 0 | 0 | 0 | 0.061 | 0 | 0.6 | 0.092 | 0.629 |
| LPAR6 | CXCL8 | 4445053 | 4438229 | 9606.ENSP00000367691 | 9606.ENSP00000306512 | 0 | 0 | 0 | 0 | 0 | 0.6 | 0.066 | 0.61 |
| NUP50 | KPNA4 | 4441567 | 4440555 | 9606.ENSP00000345895 | 9606.ENSP00000334373 | 0 | 0 | 0 | 0.062 | 0.393 | 0 | 0.361 | 0.604 |
| LCN2 | TIMP1 | 4444080 | 4433052 | 9606.ENSP00000362108 | 9606.ENSP00000218388 | 0 | 0 | 0 | 0.064 | 0 | 0 | 0.586 | 0.596 |
| CXCL8 | BCL2A1 | 4438229 | 4435793 | 9606.ENSP00000306512 | 9606.ENSP00000267953 | 0 | 0 | 0 | 0.349 | 0 | 0 | 0.4 | 0.593 |
| LCN2 | CXCL8 | 4444080 | 4438229 | 9606.ENSP00000362108 | 9606.ENSP00000306512 | 0 | 0 | 0 | 0.107 | 0 | 0 | 0.561 | 0.591 |
| IGLL5 | PAX5 | 4449581 | 4442079 | 9606.ENSP00000431254 | 9606.ENSP00000350844 | 0 | 0 | 0 | 0.102 | 0 | 0 | 0.551 | 0.579 |
| TCF7L2 | IRS1 | 4443308 | 4438085 | 9606.ENSP00000358404 | 9606.ENSP00000304895 | 0 | 0 | 0 | 0 | 0 | 0 | 0.559 | 0.559 |
| MYC | TCF3 | 4451407 | 4435174 | 9606.ENSP00000479618 | 9606.ENSP00000262965 | 0 | 0 | 0 | 0.089 | 0 | 0 | 0.534 | 0.557 |
| GNA13 | PDE4B | 4448134 | 4440326 | 9606.ENSP00000400717 | 9606.ENSP00000332116 | 0 | 0 | 0 | 0 | 0.125 | 0 | 0.511 | 0.554 |
| HP | CXCL8 | 4441808 | 4438229 | 9606.ENSP00000348170 | 9606.ENSP00000306512 | 0 | 0 | 0 | 0 | 0 | 0 | 0.553 | 0.553 |
| NR4A2 | ATF3 | 4441430 | 4441415 | 9606.ENSP00000344479 | 9606.ENSP00000344352 | 0 | 0 | 0 | 0.196 | 0 | 0 | 0.452 | 0.541 |
| MYC | PDGFRA | 4451407 | 4434540 | 9606.ENSP00000479618 | 9606.ENSP00000257290 | 0 | 0 | 0 | 0 | 0.139 | 0 | 0.475 | 0.528 |
| PDE4B | PRKAR2B | 4440326 | 4435635 | 9606.ENSP00000332116 | 9606.ENSP00000265717 | 0 | 0 | 0 | 0.146 | 0.157 | 0 | 0.393 | 0.525 |
| ATF3 | TCF3 | 4441415 | 4435174 | 9606.ENSP00000344352 | 9606.ENSP00000262965 | 0 | 0 | 0 | 0 | 0 | 0 | 0.523 | 0.523 |
| ATF3 | TIMP1 | 4441415 | 4433052 | 9606.ENSP00000344352 | 9606.ENSP00000218388 | 0 | 0 | 0 | 0.062 | 0 | 0 | 0.507 | 0.518 |
| S100A8 | SERPINE1 | 4443164 | 4433266 | 9606.ENSP00000357722 | 9606.ENSP00000223095 | 0 | 0 | 0 | 0.062 | 0 | 0 | 0.506 | 0.516 |
| CXCL2 | LCN2 | 4449392 | 4444080 | 9606.ENSP00000427279 | 9606.ENSP00000362108 | 0 | 0 | 0 | 0.098 | 0 | 0 | 0.485 | 0.515 |
| HSPA1A | MCL1 | 4444600 | 4443243 | 9606.ENSP00000364802 | 9606.ENSP00000358022 | 0 | 0 | 0 | 0.062 | 0.305 | 0 | 0.314 | 0.514 |
| ATF3 | HRK | 4441415 | 4434556 | 9606.ENSP00000344352 | 9606.ENSP00000257572 | 0 | 0 | 0 | 0 | 0 | 0 | 0.51 | 0.511 |
| MYC | IGLL5 | 4451407 | 4449581 | 9606.ENSP00000479618 | 9606.ENSP00000431254 | 0 | 0 | 0 | 0 | 0 | 0 | 0.51 | 0.51 |
| HSPA1A | ATF3 | 4444600 | 4441415 | 9606.ENSP00000364802 | 9606.ENSP00000344352 | 0 | 0 | 0 | 0.177 | 0.062 | 0 | 0.41 | 0.504 |
| PACS1 | CPD | 4439055 | 4433330 | 9606.ENSP00000316454 | 9606.ENSP00000225719 | 0 | 0 | 0 | 0 | 0 | 0 | 0.5 | 0.5 |
| MYC | IRS1 | 4451407 | 4438085 | 9606.ENSP00000479618 | 9606.ENSP00000304895 | 0 | 0 | 0 | 0 | 0 | 0 | 0.497 | 0.497 |
| CXCL8 | RIPK2 | 4438229 | 4433128 | 9606.ENSP00000306512 | 9606.ENSP00000220751 | 0 | 0 | 0 | 0.124 | 0 | 0 | 0.448 | 0.495 |
| S100A9 | CXCL8 | 4443166 | 4438229 | 9606.ENSP00000357727 | 9606.ENSP00000306512 | 0 | 0 | 0 | 0.162 | 0 | 0 | 0.42 | 0.493 |
| CXCL2 | ATF3 | 4449392 | 4441415 | 9606.ENSP00000427279 | 9606.ENSP00000344352 | 0 | 0 | 0 | 0.204 | 0 | 0 | 0.39 | 0.493 |
| HP | SERPINE1 | 4441808 | 4433266 | 9606.ENSP00000348170 | 9606.ENSP00000223095 | 0 | 0 | 0 | 0 | 0.129 | 0 | 0.44 | 0.491 |
| MCL1 | CCND2 | 4443243 | 4434845 | 9606.ENSP00000358022 | 9606.ENSP00000261254 | 0 | 0 | 0 | 0.06 | 0 | 0 | 0.481 | 0.491 |
| CXCL2 | TIMP1 | 4449392 | 4433052 | 9606.ENSP00000427279 | 9606.ENSP00000218388 | 0 | 0 | 0 | 0.089 | 0 | 0 | 0.461 | 0.488 |
| LCN2 | S100A9 | 4444080 | 4443166 | 9606.ENSP00000362108 | 9606.ENSP00000357727 | 0 | 0 | 0 | 0.227 | 0 | 0 | 0.365 | 0.488 |
| NR4A2 | CREM | 4441430 | 4435595 | 9606.ENSP00000344479 | 9606.ENSP00000265372 | 0 | 0 | 0 | 0.062 | 0 | 0 | 0.474 | 0.485 |
| ATF3 | CXCL8 | 4441415 | 4438229 | 9606.ENSP00000344352 | 9606.ENSP00000306512 | 0 | 0 | 0 | 0.101 | 0 | 0 | 0.449 | 0.483 |
| MYC | BCL2A1 | 4451407 | 4435793 | 9606.ENSP00000479618 | 9606.ENSP00000267953 | 0 | 0 | 0 | 0 | 0.064 | 0 | 0.47 | 0.482 |
| MYC | RHOBTB1 | 4451407 | 4440888 | 9606.ENSP00000479618 | 9606.ENSP00000338671 | 0 | 0 | 0 | 0 | 0.336 | 0 | 0.244 | 0.476 |
| MAFF | FOSL2 | 4441516 | 4435485 | 9606.ENSP00000345393 | 9606.ENSP00000264716 | 0 | 0 | 0 | 0.112 | 0.055 | 0 | 0.426 | 0.476 |
| IGLL5 | TCF3 | 4449581 | 4435174 | 9606.ENSP00000431254 | 9606.ENSP00000262965 | 0 | 0 | 0 | 0 | 0 | 0 | 0.475 | 0.475 |
| MYC | SERPINE1 | 4451407 | 4433266 | 9606.ENSP00000479618 | 9606.ENSP00000223095 | 0 | 0 | 0 | 0.096 | 0 | 0 | 0.44 | 0.472 |
| NR4A3 | FOSL2 | 4451630 | 4435485 | 9606.ENSP00000482027 | 9606.ENSP00000264716 | 0 | 0 | 0 | 0.101 | 0 | 0 | 0.427 | 0.463 |
| IRS1 | SERPINE1 | 4438085 | 4433266 | 9606.ENSP00000304895 | 9606.ENSP00000223095 | 0 | 0 | 0 | 0.065 | 0 | 0 | 0.449 | 0.463 |
| MAFF | NR4A2 | 4441516 | 4441430 | 9606.ENSP00000345393 | 9606.ENSP00000344479 | 0 | 0 | 0 | 0.118 | 0.157 | 0 | 0.328 | 0.457 |
| DAPK1 | CCND2 | 4447310 | 4434845 | 9606.ENSP00000386135 | 9606.ENSP00000261254 | 0 | 0 | 0 | 0.06 | 0 | 0 | 0.446 | 0.456 |
| GTSE1 | SAC3D1 | 4448783 | 4446865 | 9606.ENSP00000415430 | 9606.ENSP00000381824 | 0 | 0 | 0 | 0.061 | 0 | 0 | 0.439 | 0.451 |
| HP | TIMP1 | 4441808 | 4433052 | 9606.ENSP00000348170 | 9606.ENSP00000218388 | 0 | 0 | 0 | 0 | 0 | 0 | 0.45 | 0.45 |
| GNA13 | CD79B | 4448134 | 4446076 | 9606.ENSP00000400717 | 9606.ENSP00000376544 | 0 | 0 | 0 | 0 | 0 | 0 | 0.45 | 0.45 |
| NR4A3 | ATF3 | 4451630 | 4441415 | 9606.ENSP00000482027 | 9606.ENSP00000344352 | 0 | 0 | 0 | 0.178 | 0 | 0 | 0.359 | 0.45 |
| ID4 | TCF3 | 4445105 | 4435174 | 9606.ENSP00000367972 | 9606.ENSP00000262965 | 0 | 0 | 0 | 0 | 0.117 | 0 | 0.4 | 0.447 |
| LCN2 | S100A8 | 4444080 | 4443164 | 9606.ENSP00000362108 | 9606.ENSP00000357722 | 0 | 0 | 0 | 0.208 | 0 | 0 | 0.329 | 0.446 |
| CREM | FOSL2 | 4435595 | 4435485 | 9606.ENSP00000265372 | 9606.ENSP00000264716 | 0 | 0 | 0 | 0.062 | 0 | 0 | 0.434 | 0.446 |
| S100A9 | HP | 4443166 | 4441808 | 9606.ENSP00000357727 | 9606.ENSP00000348170 | 0 | 0 | 0 | 0.084 | 0 | 0 | 0.41 | 0.436 |
| CCND2 | PDGFRA | 4434845 | 4434540 | 9606.ENSP00000261254 | 9606.ENSP00000257290 | 0 | 0 | 0 | 0.051 | 0.142 | 0 | 0.358 | 0.431 |
| FGF12 | PDGFRA | 4448693 | 4434540 | 9606.ENSP00000413496 | 9606.ENSP00000257290 | 0 | 0 | 0 | 0 | 0.267 | 0 | 0.256 | 0.431 |
| PAX5 | RIPK2 | 4442079 | 4433128 | 9606.ENSP00000350844 | 9606.ENSP00000220751 | 0 | 0 | 0 | 0 | 0.087 | 0 | 0.401 | 0.43 |
| MYC | MECP2 | 4451407 | 4447894 | 9606.ENSP00000479618 | 9606.ENSP00000395535 | 0 | 0 | 0 | 0 | 0 | 0 | 0.427 | 0.427 |
| ADM | CXCL8 | 4449788 | 4438229 | 9606.ENSP00000436607 | 9606.ENSP00000306512 | 0 | 0 | 0 | 0.088 | 0 | 0 | 0.397 | 0.426 |
| MYC | SPAG9 | 4451407 | 4434990 | 9606.ENSP00000479618 | 9606.ENSP00000262013 | 0 | 0 | 0 | 0 | 0.336 | 0 | 0.172 | 0.426 |
| MYC | TIMP1 | 4451407 | 4433052 | 9606.ENSP00000479618 | 9606.ENSP00000218388 | 0 | 0 | 0 | 0.081 | 0 | 0 | 0.398 | 0.423 |
| TCF7L2 | CCND2 | 4443308 | 4434845 | 9606.ENSP00000358404 | 9606.ENSP00000261254 | 0 | 0 | 0 | 0.06 | 0 | 0 | 0.406 | 0.417 |
| MYC | ITGA6 | 4451407 | 4447442 | 9606.ENSP00000479618 | 9606.ENSP00000386896 | 0 | 0 | 0 | 0.062 | 0 | 0 | 0.403 | 0.416 |
| MYC | HSPA1A | 4451407 | 4444600 | 9606.ENSP00000479618 | 9606.ENSP00000364802 | 0 | 0 | 0 | 0.051 | 0.111 | 0 | 0.36 | 0.413 |
| LCN2 | SERPINE1 | 4444080 | 4433266 | 9606.ENSP00000362108 | 9606.ENSP00000223095 | 0 | 0 | 0 | 0.061 | 0 | 0 | 0.398 | 0.41 |
| MAPK7 | FOSL2 | 4438648 | 4435485 | 9606.ENSP00000311005 | 9606.ENSP00000264716 | 0 | 0 | 0 | 0 | 0.264 | 0 | 0.227 | 0.407 |
| SLC2A14 | IRS1 | 4450179 | 4438085 | 9606.ENSP00000445929 | 9606.ENSP00000304895 | 0 | 0 | 0 | 0 | 0 | 0 | 0.406 | 0.406 |
| ID4 | TCF7L2 | 4445105 | 4443308 | 9606.ENSP00000367972 | 9606.ENSP00000358404 | 0 | 0 | 0 | 0 | 0 | 0 | 0.406 | 0.406 |
| ADM | SERPINE1 | 4449788 | 4433266 | 9606.ENSP00000436607 | 9606.ENSP00000223095 | 0 | 0 | 0 | 0.152 | 0 | 0 | 0.327 | 0.405 |
| MYC | SLC2A3 | 4451407 | 4432652 | 9606.ENSP00000479618 | 9606.ENSP00000075120 | 0 | 0 | 0 | 0 | 0 | 0 | 0.404 | 0.404 |
| MYC | CD79B | 4451407 | 4446076 | 9606.ENSP00000479618 | 9606.ENSP00000376544 | 0 | 0 | 0 | 0.049 | 0 | 0 | 0.398 | 0.403 |
| S100A8 | HP | 4443164 | 4441808 | 9606.ENSP00000357722 | 9606.ENSP00000348170 | 0 | 0 | 0 | 0.081 | 0 | 0 | 0.375 | 0.402 |
| HSPA1A | CREM | 4444600 | 4435595 | 9606.ENSP00000364802 | 9606.ENSP00000265372 | 0 | 0 | 0 | 0.049 | 0.261 | 0 | 0.218 | 0.402 |
| SPRY1 | FGF12 | 4451603 | 4448693 | 9606.ENSP00000481675 | 9606.ENSP00000413496 | 0 | 0 | 0 | 0 | 0 | 0 | 0.402 | 0.402 |
| NR4A3 | NR4A2 | 4451630 | 4441430 | 9606.ENSP00000482027 | 9606.ENSP00000344479 | 0 | 0 | 0.951 | 0.376 | 0 | 0 | 0.792 | 0.4 |

Supplement Table2. The overlapping target genes authenticated by miRwalk and TargetScan database.

| miRNA | Target genes |
| --- | --- |
| hsa-miR-142-3p  (n=64) | RRAS2, CPEB2, ZFYVE16, NAB1, HNRNPK, IL1A, ACADL, ITGA4, FMR1, SPRED1, STAU2, RALGPS2, CLTA, NUFIP2, EIF5, PAM, NFKBIE, GREM1, FYCO1, C6orf62, C2CD2, OTUD4, SLA, UBE2W, MTMR9, SLC35F1, APBB2, ACSL1, TOR1AIP2, TLCD2, AKT3, ATG16L1, ZBTB37, DCLK1, ZMIZ2, RYBP, RIC8B, SFMBT1, LPP, CANX, GRIN2B, WNK3, VCL, PDE12, UBN2, ARHGAP5, MTF1, EMB, PCDH17, CRTC3, MED13, MAP4K4, HDAC2, FBXO3, PDE4B, KCNS2, NSF, ZZZ3, SOCS6, ANKS1A, AGO2, ZNF618, XIAP, MEF2C |
| hsa-miR-17-5p  (n=194) | NAGK, ENPP5, PKD2, SERP1, FBXO48, MYT1L, FAM219B, FGD4, RRAGD, KCNK10, CDC37L1, CD69, PLSCR4, NR4A3, MAPRE3, ANKRD52, CRYBG3, UEVLD, SAMD12, CEP128, CCNG2, BMPR2, TBC1D17, SLC24A2, CALD1, ORMDL3, RPS6KA5, AHNAK, PPP6C, COX7A2L, ZNF2, KIF5A, CADM2, LDLR, RGMA, SMIM14, AGFG2, LHX6, EPHA5, ITGB8, TMEM64, GNS, DPYSL2, ZC3H12C,GAB1, LYPD6, GNB5, SCN1A, CRY2, ZBTB41, RBL2, SLC16A9, UBXN2A, LZIC, IRF1, TTPAL, ERI1, WDR37, TNFSF11, NEDD4L, NPAT, ETV1, BNIP2, NHLRC3, ESR1, ABL2, MFSD9, HPS5, CNGB3, ATE1, DPYSL5, OSTM1, METAP1, CYP2U1, BAHD1, TNKS2, LRRC55, ZNF704, MAP3K9, ERBB3, DGKH, RAPGEFL1, TCF4, APP, MTF1, SHOC2, EIF4G2, DCUN1D3, LPGAT1, TAOK1, PPP1R3B, FAM189A1, POLQ, SLC45A4, KCNJ10, RAB11FIP1, FRMD4B, FBXO21, MTMR3, HMGB3, WEE1, ATP1A2, UBE3C, ATXN1L, TBCEL, RND3, RABEP1, NEK9, SMAD5, SLC39A6, ANKRD13C, SLITRK2, ZFYVE26, PLEKHM1, DIP2A, ATXN1, TLE4, PKN2, HOOK3, TMEM168, KIF3B, RAB5B, HEG1, KIAA1671, AGO1, ZDHHC8, DCBLD2, AAK1, FSD1L, CCND2, ENTPD4, ZBTB8A, RAB30, ZNF805, WDFY3, VEGFA, GOSR1, RGS4, WNT9B, FLT1, KLF9, MCM3, INO80, PDLIM5, DSTYK, PGM2L1, FNDC3A, C16orf72, RASGRF2, RPS6KA2, IPO9, SLC24A4, ALX4, MYLIP, PDK3, CYB561D1, ARHGAP26, PRDM10, MBD5, RNF217, FGF5, UBASH3B, RAB11FIP4, WNK3, DOK6, ACTR1A, ROCK2, AFF4, C16orf70, SLC25A36, E2F3, ARSJ , TNRC6B, MKLN1, PHC3, ARMC8, XIAP, NFAT5, ABCA1, ZNF652, AK4, ATG14, BRWD1, TMEM50B, TANC2, PMK, DNAL1, CCSER2, TUSC2, RPS6KA6, ANKH, LRP8, ZBTB20, COL19A1 |

Supplement Table3. Genes overlapped between the target genes of the TFs and DEGs.

| TF | Overlapping genes |
| --- | --- |
| ATF3  (n=96) | SAC3D1, NR4A3, SRSF8, ORAI2, CD58, BCL2A1, GNA13, MAPK7, ACSL1, NR4A2, CDC42EP3, CREM, SLC39A8, ADM, FOSL2, PDE4B, LCN2, CPD, GTSE1, MAFF, MARCKS, RPL35A, HSPA1A, HSPA1B, CCND2, MRPS12, TCF3, NEU1, CD79B, ZNF165, NRTN, SPAG9, RFWD3, SERPINE1, CXCL11, AEBP1, BASP1, DAPK1, RIPK2, SLC2A3, PACS1, IRS1, CHST2, EIF5, TIMP1, MCL1, HRK, MYC, HCAR3, ELF4, PON2, KIAA0226L, RECQL5, NUP50, RPL37A, SPRY1, LHPP, NUDT6, PAX5, TBL1X, GCH1, VASH2, KPNA4, PRG2, IQCK, PDGFRA, CHN2, BAHCC1, S100A8, HNRNPM, STAB1, HP, FERMT2, TMSB15B, TCF7L2, LPAR6, SLC2A14, S100A9, OLFM4, TMSB15A, FGF12, RHOBTB1, SPON1, EMP1, PCDH9, PTPRM, ZNF611, ITGA6, SOX11, TMCC1, CXCL2, SLIT3, ID4, PRKAR2B |
| CREM  (n=96) | SERPINE1, CXCL2, MCL1, ACSL1, EMP1, ADM, MAFF, PON2, RHOBTB1, ATF3, SPAG9, RECQL5, MYC, SLC2A14, CPD, FOSL2, EIF5, ITGA6, MAPK7, LCN2, PACS1, TCF7L2, TBL1X, BASP1, KPNA4, PRKAR2B, CDC42EP3, PDE4B, NRTN, PTPRM, TMCC1, ORAI2, HRK, S100A8, HNRNPM, SPON1, IQCK, MECP2, NUDT6, S100A9, PRG2, BAHCC1, SPRY1, SAC3D1, RFWD3, CHN2, KIAA0226L, VASH2, FERMT2, IRS1, HCAR3, SLC2A3, NEU1, SLIT3, RIPK2, GNA13, NR4A3, NR4A2, SLC39A8, CD58, AEBP1, GTSE1, NUP50 ,HSPA1B, DAPK1, FGF12, MARCKS, TCF3, HSPA1A, LHPP, SRSF8, CCND2, LPAR6, OLFM4, PCDH9, GCH1, BCL2A1, HP, CD79B, ZNF611, MRPS12, RPL37A, SOX11, RPL35A, CHST2, STAB1, CXCL11, PDGFRA, ID4, ZNF165, PAX5, ELF4, TIMP1, TMSB15A, TMSB15B |
| ELF4  (n=96) | MAFF, SPAG9, ATF3, SAC3D1, MRPS12, LCN2, ORAI2, NEU1, GTSE1, HSPA1A, HNRNPM, TCF3, GNA13, TMCC1, MAPK7, LHPP, NUP50, RECQL5, HSPA1B, SERPINE1, SLC39A8, PRKAR2B, KPNA4, ZNF165, SLIT3, RPL35A, CCND2, PACS1, PDGFRA, SPON1, CD79B, HRK, DAPK1, FERMT2, STAB1, S100A8, TMSB15B, RFWD3, CREM, VASH2, HCAR3, NRTN, PRG2, CDC42EP3, PAX5, EIF5, S100A9, CPD, BAHCC1, TIMP1, CD58, FGF12, MYC, OLFM4, ADM, IRS1, PDE4B, CHN2, ACSL1, AEBP1, RPL37A, LPAR6, SLC2A3, ZNF611, KIAA0226L, MCL1, HP, NUDT6, EMP1, TCF7L2, RHOBTB1, SRSF8, SLC2A14, PCDH9, GCH1, BCL2A1, IQCK, PTPRM, ITGA6, SOX11, FOSL2, NR4A2, CHST2, SPRY1, CXCL2, CXCL11, BASP1, ID4, MARCKS, PON2, RIPK2, NR4A3, MECP2, TBL1X, TMSB15A |
| FOSL2  (n=96) | HSPA1A, HSPA1B, BAHCC1, NEU1, TCF3, TIMP1, ZNF611, MCL1, NRTN, IQCK, RFWD3, SLC2A3, SAC3D1, MRPS12, SPAG9, HRK, LCN2, CREM, GTSE1, CHST2, DAPK1, RPL37A, SERPINE1, PON2, EIF5, ZNF165, LHPP, MARCKS, MAPK7, SLC39A8, RECQL5, NUDT6, RPL35A, NUP50, KPNA4, SPRY1, SRSF8, TMCC1, NR4A3, CXCL11, HP, TBL1X, MAFF, ELF4, AEBP1, HNRNPM, PACS1, ATF3, S100A9, ACSL1, CHN2, SLC2A14, IRS1, MECP2, ORAI2, CXCL2, CPD, HCAR3, PAX5, PRG2, GNA13, S100A8, BCL2A1, LPAR6, PRKAR2B, VASH2, ADM, TMSB15B, CD79B, CD58, TMSB15A, EMP1, NR4A2, PDE4B, TCF7L2, RHOBTB1, SPON1, CCND2, KIAA0226L, OLFM4, PCDH9, GCH1, FERMT2, PTPRM, ITGA6, CDC42EP3, SOX11, FGF12, STAB1, PDGFRA, BASP1, SLIT3, ID4, MYC, RIPK2 |
